# The Effects of a Cold Disinfestation on *Bactrocera dorsalis* Survival and Navel Orange Quality

**DOI:** 10.3390/insects10120452

**Published:** 2019-12-13

**Authors:** Yan Fang, Fenfen Kang, Guoping Zhan, Chen Ma, Yuguang Li, Lin Wang, Yadong Wei, Xiwu Gao, Zhihong Li, Yuejin Wang

**Affiliations:** 1 Department of Entomology, College of Plant Protection, China Agricultural University, Beijing 100193, China; yanfang_926@163.com (Y.F.); kangfenfen@customs.gov.cn (F.K.); lyg727493751@163.com (Y.L.); SY20183192482@cau.edu.cn (L.W.); gaoxiwu@263.net.cn (X.G.); lizh@cau.edu.cn (Z.L.); 2 The Animal, Plant & Foodstuff Inspection Center, Tianjin Customs, Tianjin 300461, China; weiyadong@customs.gov.cn; 3 Chinese Academy of Inspection and Quarantine, Beijing 100123, China; gp136@126.com; 4 National Agro-Tech Extension and Service Center, Beijing 100125, China; macfov@hotmail.com

**Keywords:** *Bactrocera dorsalis*, navel orange, ecofriendly pest management, mortality, fruit quality

## Abstract

*Citrus sinensis* (L.) Osbeck is an important economic product in South China, but the presence of quarantine pests in this product proposes the potential threat to international trade security. To find a proper phytosanitary cold treatment for *Bactrocera dorsalis* (Hendel) (Diptera: Tephritidae)*,* commonly called oriental fruit fly, one of the most serious quarantine insects in navel orange, eggs in petri dish and larvae in navel orange fruits were exposed to a 1.7 °C cold chamber for 0–11 days to compare the tolerance to cold treatment. The 2nd instar larva (4 days) is the most tolerant stage, and the estimated time for 99.9968% mortality at the 95% confidence level is 11.3 (9.5, 14.6) days. Then 15 days was selected as the target time for the confirmatory tests, resulting in no survivors from 37,792 treated larvae with the efficacy of 99.9921% mortality at the 95% confidence level. The quality assessments were conducted to compare the effect on the navel orange fruit between cold treatment and the conventional cold storage. Results indicated that the cold treatment did not negatively affect the fruit quality. Therefore, this cold treatment showed potential as a commercial quarantine treatment for navel orange in international trade.

## 1. Introduction

The oriental fruit fly, *Bactrocera dorsalis* (Hendel), is one of the world’s most damaging pests and causes trade restrictions on fresh fruits [1,2,3]. This pest has previously been recorded to have a wide range of hosts with more than 400 plant species in a list published by the United States Department of Agriculture–Animal and Plant Health Inspection Service (USDA-APHIS) [4]. *B. dorsalis* is a highly invasive species and is an important quarantine pest in Asia and Pacific Plant Protection Commission (APPPC), European Plant Protection Organization (EPPO), Caribbean Plant Protection Commission (CPPC), Comité de Sanidad Vegetal del Cono Sur (COSAV), Inter-African Phytosanitary Council (IAPSC), and Organismo Internacional Regional de Sanidad Agropecuaria (OIRSA). As of now, the fruit fly has been reported in at least 65 countries, including most of Asia, much of sub-Saharan Africa, parts of America, Oceania, and Europe [5,6,7,8]. The navel orange (“Gannan” variety), *Citrus sinensis* (L.) Osbeck, is famous worldwide for its high quality. In 2018, the navel orange industry achieved a total output value of nearly 2 billion dollars in China and outside markets, employed 1 million rural labor workers on 104,000 ha that produced 1,170,000 tons [9]. Unfortunately, *B. dorsalis* is a potential pest in the navel orange, threatening the safety of the navel orange export [5].

The risk of introducing exotic pests into new areas is rising in the wake of the increasing agricultural trade, and the development of postharvest treatment to control insects in commodities can improve the quarantine security and expedite new trade in agricultural products. The main pest management approaches for eliminating exotic insects from a commodity are applied broadly as chemical and physical treatments. These include fumigants, temperature treatments (heat and cold), irradiation, controlled atmospheres, and combinations of these [10]. Methyl bromide fumigation is the most common approach for controlling exotic pests but is expected to be replaced by physical treatments because of methyl bromide depleting the ozone layer [10]. Physical treatments are typically applied to fresh fruits because these commodities are infested by internally feeding pests, such as tephritid fruit flies, and cold treatment is an effective measure in cold-tolerant commodities [11,12]. The pupa and adult stages of tephritid fruit flies may hardly be found in infested fruit, and phytosanitary treatments target the egg and larval stages for disinfestation [13].

Research studies have demonstrated that cold disinfestation is an effective approach in controlling *Bactrocera zonata* (Saunders) in orange [14,15]; *Bactrocera invadens* (Drew, Tsuruta, and White) (synonym of *B. dorsalis*) in orange, and “Hass” avocado [15,16,17]; *Zeugodacus cucurbitae* (Coquillett) (former *Bactrocera cucurtitae* (Coquillett)) in navel orange [13]; *Bactrocera tryoni* (Froggatt) in lemon, orange, mandarin, and blueberry [18,19,20]; *Ceratitis capitata* (Wiedemann) in kiwifruit, litchi, lemon, orange, mandarin, grape, pepper, “Hass” avocado, and navel orange [13,20,21,22,23,24,25,26]; *Ceratitis rosa* (Karsch) in “Hass” avocado [26]; *Ceratitis cosyra* (Walker) in “Hass” avocado [26]; *Phthorimaea operculella* (Zeller) in potato [27]; and *Pseudococcus affinis* (Maskell) on Royal Gala Apple [28]. The effective lethal time value for cold disinfestation was dependent on the type of insect and fruit [29]. Furthermore, earlier researches have compared the difference of the tolerance to cold treatment between fruit flies [13,30,31]. Until now, many cold treatment standards have been widely used internationally [32,33].

Traditionally, a treatment protocol should provide quarantine security for a pest with minimal impact on commodity quality [10,34]. An integrated quarantine treatment first with exposure to 1.5 °C for 3 days and second with exposure to a controlled atmosphere at 25 °C could complete insect mortality with no negative effects on the quality of “Clemenules” mandarins [35]. Hofman et al. reported that an integrated treatment with hot water of 41 °C for 25–30 min, or 42 °C for 25 min following 16 days at 1 °C, could improve the external and internal fruit quality of avocado and control fruit flies [36]. Other data showed that 6 °C for 3 days, followed by 1 °C for 16 days, improves ripe “Hass” avocado fruit quality, with no negative effects, while also providing *B. tryoni* disinfestation efficacy [37]. Among the treatments accepted for quarantine measures, cold disinfestation is an effective approach in controlling pests that are present in fresh fruits [32,33]. However, the treatment also needs to meet the requirements for minimal impact on commodity quality.

The objective of this study was to compare the cold tolerance of the different life stages of *B. dorsalis* that infest navel orange and determine the minimum number of days of cold treatment that provides quarantine security with no negative effects on navel orange quality.

## 2. Materials and Methods

### 2.1. Test Insects

The original population of *B. dorsalis* that was used in this experiment was collected from wax apple in Chongzuo, China, in 2017 and 2018. This species is maintained at the Chinese Academy of Inspection and Quarantine in Beijing, China, and reared on a standard diet modified from Liu et al. [38]. This fruit fly colony has been maintained for nearly 1 year with the occasional inclusion of wild flies.

### 2.2. Fruit Infestation

The navel oranges, (“Gannan” variety) originating from the Gannan area, were sourced from Jinyuanbao Binhai agricultural product transaction market in Tianjin, China, and were stored at 4.0 °C. The fruits did not receive direct pesticide applications and were free from pesticide residues. To reduce contamination with microorganisms, the fruits (mean weight = 0.22 ± 0.03 kg) were washed with dishwashing liquid, rinsed with water, and then allowed to air dry at ambient temperature.

The navel orange is an attractive host for *B. dorsalis*, and larvae were obtained by puncturing fruits with pins and exposing them to adults in screen cages (120 by 60 by 60 cm) for oviposition. The age and number of the adult flies in each cage could influence the number of eggs in each fruit. On each of the infestation, 63 fruits were placed in a cage which contains about 8000 adults (rate of male/female is nearly 1:1). The infestation was conducted 15–30 days after adult emergence and infested 3–5 times in one batch of the colony. To obtain an infestation rate of >50 larvae per fruit, each fruit was punctured approximately 50 times to a depth of 1–2 cm and was exposed to fruit flies in the cages for 2–4 h. Eggs in fruits were incubated at 26 ± 0.5 °C for 2, 4, and 6 days to obtain the 1st, 2nd, and 3rd instars, respectively. In the egg trial, eggs were collected from egging bottles, then pipetted onto pieces of black cotton cloth and placed in a 0.1 m petri dish with moderate distilled water and 3 drops of sugar water for hatching in the experiments. Each petri dish contained 200 eggs.

### 2.3. Development of B. dorsalis in the Navel Orange

Navel oranges infested with fruit fly were stored to 26 ± 0.5 °C and 65% ± 5% RH for various periods. In this study, we controlled development time to affect their capacity to develop into the different larval stages. To obtain enough larvae, fruits were exposed to fruit flies in the cages for approximately 2 h. Fruits in ventilated plastic cages were placed in a climate chamber (KBF 720, BINDER Inc, Tuttlingen, Germany) with a stated accuracy of 0.5 °C. At days 2, 4, 6, 8, and 9, approximately 100 larvae in 2–3 fruits infested with *B. dorsalis* were randomly selected and immediately examined with a dissecting microscope for them in each treatment. According to the identification from Zhou et al., the stages of *B. dorsalis* larvae were recorded [39].

### 2.4. Effect of Cold Disinfestation on B. dorsalis Survival

Different larval stages of *B. dorsalis* in the navel oranges were exposed to the cold chamber for various periods to examine the effect of cold disinfestation on pest survival. Fruits infested with *B. dorsalis* were placed on a rectangular plastic basket and loaded into a chamber (HRT-105P, Chong Qing Well Co, Chongqing, China) with an accuracy of 1.7 °C ± 0.3 °C for cold treatment. Before each trial, the thermoprobes were calibrated by a calibrator (RTC-156 B, Ametek Co., Alleroed, Denmark). Each test read 10 times, and the average result for each thermoprobe was used for correction purposes. During the experiment, thermoprobes (Testo 177-T4, Testo SE & Co. KGaA, Lenzkirch, Germany) with an accuracy of ± 0.2 °C were placed at a diagonal in the core of the largest fruit in the environmental chamber, and six temperatures of the chamber and the center of fruit were recorded every 30 min.

In the egg treatment, the petri dish temperatures were consistent with the chamber air temperature, and the treatment was deemed to have started immediately after the chamber air temperature reached the target treatment temperature. Larvae treatment was deemed to have started immediately after all the centers of the fruits had reached the target temperature. In this study, the center temperature of the fruits required about 4 h to reach the target temperature. On days 0, 1, 3, 5, 7, 9, and 11, petri dishes loaded with eggs and fruits infested with *B. dorsalis* were randomly selected, removed from the cold chamber, and examined for mortality after 5 days and 2 days recovery in the 26.0 °C chamber. Three replicates were used in the larval trial and four in the egg trial. Eggs that were hatching were counted as live, and larvae that did not move were scored as dead. Larvae movement or not was determined by placing the unmoving larvae on a napkin.

### 2.5. Confirmatory Tests

Based on the results of the effect of cold disinfestation on survival to determine which life stage of *B. dorsalis* was the most cold-tolerant, a large-scale disinfestation trial was undertaken over the most cold-tolerant life stage of this pest. On each of the confirmatory tests, 54 navel oranges were placed in the cage for oviposition. Approximately 90% of the fruits infested with *B. dorsalis* were placed into a 1.7 °C cold chamber (70 by 80 by 90 cm), and the remaining infested fruits were used as the control. During the confirmatory tests, thermoprobes were placed at a diagonal in the core of the largest fruit in the cold chamber. Treatment was deemed to have begun, and mortality of larvae was determined as above.

### 2.6. Fruit Quality Assessment

Navel orange fruits were used for the assessment of the internal and external qualities at cold treatment (1.7 °C) and the conventional cold storage (4 °C). The changes in fruit quality were compared between cold treatment and cold storage. In the case of cold treatment, the fruit quality was determined on the fruits after exposure to 1.7 °C for 0, 8, and 15 days, followed by a 7 day cold storage period at 4 °C. In the case of cold storage treatments, the fruit quality was determined for the fruits after the exposure to 4.0 °C for 0, 8, 15, and 22 days. The following quality attributes were determined: peel color, fruit firmness, vitamin C (Vc), soluble solids concentration (SSC), and titratable acidity (TA).

#### 2.6.1. Peel Color

The peel color was determined by Hunter Lab parameters with a colorimeter (Chroma meter CR-400, Konica Minolta sensing Inc., Tokyo, Japan). A specific color index (CI) for citrus was calculated as CI = 1000 × a/(L × b) [40]. For each treatment, 36 fruits were measured three times each, along the equatorial region of each fruit.

#### 2.6.2. Fruit Firmness

The firmness of 36 fruits was evaluated by using a penetrometer (Fruit pressure tester FT327, TR Ltd., Forli, Italy) equipped with a 3 mm diameter probe and operated at a constant penetration speed, with each fruit being measured 3 times along the equatorial region.

#### 2.6.3. Vitamin C

For each treatment, vitamin C (Vc) was extracted from fresh navel orange flesh using oxalates and then filtered for use. Three measures of vitamin C were determined in 9 different fruits per treatment. Vitamin C was quantified by titration with 2 6-dichlorophenol indophenol, and the results were recorded as grams of vitamin C per kilogram of navel orange flesh.

#### 2.6.4. Soluble Solids Concentration

The soluble solids concentration (SSC) was determined by using a digital refractometer (RX-5000α, ATAGO Co. LTD., Tokyo, Japan) at 20 °C and expressed as a percentage. For each treatment, 12 fruits comprising 6 replications were squeezed to obtain juice.

#### 2.6.5. Titratable Acidity

For the experiments, the titratable acidity (TA) was determined by titration with 0.1 mol/L NaOH with a 1% phenolphthalein indicator (% w/v), and the results were recorded as a percentage (% w/w). For each treatment, the juice was obtained from 9 fruits, and each measurement comprised three replications.

### 2.7. Statistical Analysis

To make comparisons among the cold tolerance of the different life stages, time–response data on the mortality were arcsine transformed to improve normality and subjected to analysis of covariance (ANCOVA) by using the General Linear Model (GLM) (version 17, SPSS Inc., Chicago, IL, USA). For each replicate, the mortality values were adjusted for control mortality calculated using Abbott’s formula [41]. Probit and logit analysis (LeOra Software, Berkeley, CA, USA) were performed on the time-response data for all days for *B. dorsalis* to calculate the predicted times to 99% (LT_99_) and 99.9968% (LT_99.9968_) mortality. For the confirmatory test, the mortality proportion (1-*Pu*) associated with treating a number of *B. dorsalis* with zero survivors was calculated by the following equation:

$$1-Pu={\left(1-C\right)}^{1/n}$$

where *C* is the confidence level, and n is the number of insects treated with no survivor [42].

To determine the effect of cold treatment and cold storage on fruit quality, two-way analysis of variance and Tukey’s multiple comparison tests were applied with the SPSS software package.

## 3. Results

### 3.1. Development of B. Dorsalis in Navel Orange

**Figure 1:**
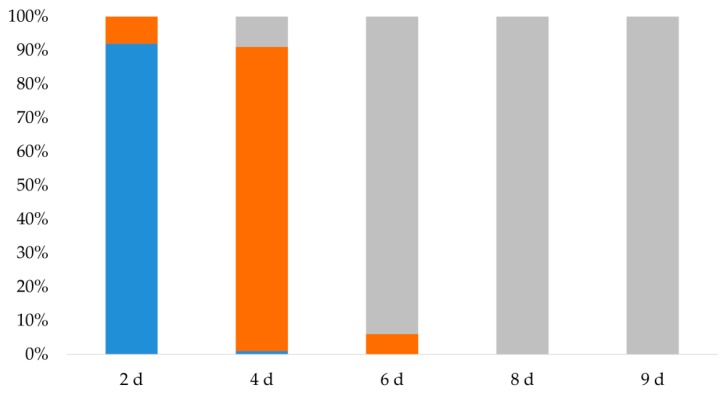
Percentage of different larval stages of *Bactrocera dorsalis* in navel orange (■, First instar; ■, Second instar; ■, Third instar).

Before the trial, the development of *B. dorsalis* larvae in navel orange was observed, Figure 1. For *B. dorsalis* in navel orange, the percentage of larval development was 92% at the 1st instar on day 2, 90% at the 2nd instar on day 4, and 94% at the 3rd instar on day 6. On days 8 and 9, all of the species tested were able to develop to 3rd instar. Therefore, we considered 2 day-old, 4 day-old, and 6 day-old larvae of this species as the 1st, 2nd, and 3rd instar, respectively.

### 3.2. Effect of Cold Disinfestation on B. Dorsalis Survival

The mortality increased with increasing treatment time for all life stages, no eggs hatched ≥7 days, and no larvae survived ≥9 days, Table 1. Result showed no significant effect of the interaction between treatment days and stages (*F*_2,30_ = 1.897; *p* = 0.168) on the survival; whereas a significant impact was found for the treatment days (*F*_1,32_ = 242.260; *p* < 0.001) and life stages (*F*_2,32_ = 8.451; *p* = 0.001). The mortality of 1st and 2nd instar larvae was significantly lower than the mortality of the 3rd instar and eggs. However, no significant difference was observed between the 1st and 2nd instar larvae. In addition, the mortality of 2nd instar was lower than that of the 1st instar at 1, 3, 5, and 7 days, Table 1.

The parameters from the results of the probit analysis (probit and logit model), including slope and estimated day to 99% (LT_99_) and 99.9968% (LT_99.9968_) mortality, are presented in Table 2. The estimated LT_99_ was very close to the day that produced 100% mortality in the cold treatment, Table 1 and Table 2, and the LT_99_ of the 2nd instar was higher than that for the other stage, indicating that the resistance of 2nd instar was higher. Furthermore, the LT_99.9968_ of the 2nd instar was highest in all tested stages. Thus, the 2nd instar larvae likely to be found in fruits were determined to be the most tolerant stage in the navel orange.

### 3.3. Confirmatory Tests

Confirmatory tests were conducted to validate the estimated day to achieve 99.9968% mortality in *B. dorsalis* 2nd instar, and 15 days was selected as the target day according to the estimates of probit and logit analysis, Table 2.

No survivors were found in the 15-day treatment at 1.7 °C, and the data are presented in Table 3, whereas the percentage of survivors in the control was 94.09%. In this study, calculating the disinfestation efficacy (1-*Pu*) with a confidence level of 95% by the equation is 99.9916%.

### 3.4. Quality of Navel Orange Exposed to Cold Treatment

**Figure 2:**
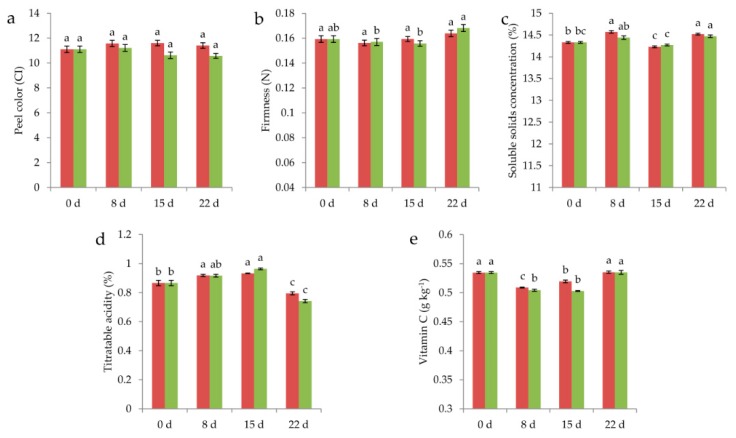
Changes in quality of navel orange, peel color (**a**), firmness (**b**), soluble solids concentration (**c**), titratable acidity (**d**) and vitamin C (**e**), through time after storage at 1.7 °C and 4.0 °C (■, 1.7 °C; ■, 4.0 °C). Data are the means ± SE. Values with different letters on each bar in the two groups differ significantly (*p* < 5%).

Comparisons of the effect of storage time and temperatures at 1.7 °C and 4.0 °C on the objective attributes of navel orange are presented in Figure 2a–e. The results showed no significant effect of the interaction between temperatures and storage time for peel color (*F*_3, 856_ = 1.721, *p* = 0.161) Figure 2a and firmness (*F*_3, 856_ = 0.836, *p* = 0.474) Figure 2b, whereas a significant impact was found for the SSC (*F*_3, 40_ = 3.852, *p* = 0.016) Figure 2c, TA (*F*_3, 16_ = 4.263, *p* = 0.022) Figure 2d, and Vc (*F*_3, 16_ = 7.707, *p* = 0.002) Figure 2e.

When the effect of storage time on the quality of the navel orange was measured, a significant impact was observed on the firmness (*F*_3, 856_ = 5.743, *p* = 0.001) Figure 2b, SSC (*F*_3, 40_ = 45.659, *p* < 0.001) Figure 2c, TA (*F*_3, 16_ = 87.157, *p* < 0.001) Figure 2d, and Vc (*F*_3, 16_ = 119.006, *p* < 0.001) Figure 2e, whereas no significant impact was observed on the peel color (*F*_3, 856_ = 0.984 *p* = 0.400) Figure 2a. The result indicated that the quality of the navel orange was affected by the storage time.

No significant difference between temperatures was observed on the firmness (*F*_1, 856_ = 0.041, *p* = 0.839) Figure 2b, SSC (*F*_1, 40_ = 3.517, *p* = 0.068) Figure 2c, and TA (*F*_1, 16_ = 0.531, *p* = 0.477) Figure 2d, whereas a significant impact was observed on the peel color (*F*_1, 856_ = 9.999, *p* = 0.002) Figure 2a and Vc (*F*_1, 16_ = 14.593, *p* = 0.002) Figure 2e. In addition, the values of CI and Vc of the navel orange changed slower in the cold treatment Figure 2a,e.

## 4. Discussion

In the present study, *B. dorsalis* 2nd instar larvae appear to be the most cold-tolerant life stages tested at 1.7 °C. Therefore, large-scale confirmatory testing for a cold disinfestation treatment targeted the 2nd instar larvae. In the confirmatory test, no survivors were found after the 15 days treatment at 1.7 °C, resulting in a disinfestation efficacy of 99.9916% at the 95% confidence level. The results suggest that holding the fruit center temperature at ≤1.7 °C for a minimum of 15 days is an effective treatment for the control of *B. dorsalis* in the navel orange.

The fruit type may affect the cold treatment period required for disinfestation. Fruit type could affect the development of fruit flies, which in turn affects the cold tolerance of fruit flies. Generally, individuals of a certain age in days are considered as the 1st, 2nd, and 3rd instars in a cold treatment study [17,26,31,43]. In this study, the percentage of larvae was 1% at 1st instar, 90% at 2nd instar, and 9% at 3rd instar on day 4, and the result was consistent with the research in oranges and “Hass” avocado [16,17]. The relationship between the percentages of the different stages and cold tolerance is not clear. Therefore, further experiments are needed to confirm the effect of the proportions of 1st, 2nd, and 3rd instars on pest cold tolerance.

The infestation method may also affect the cold treatment period required for disinfestation. Commonly, a natural infestation method and an artificial infestation method are the two main types of infection methods [13,15]. In artificially infested fruit, eggs are placed near the fruit center but are laid near the fruit surface in naturally infested fruit. The fruit center is the warmest place during the treatment, differences in the infestation methods may have an influence on the response to the cold treatment. In addition, the temperature, most tolerant life stage, colony source, and the identified endpoint were considered the factors that affect the cold treatment period required for disinfestation [13].

Usually, an effective cold treatment protocol should meet the demand to kill the pest and minimize the negative effects on host quality at the same time [10,29,34]. The present results confirm that cold treatment did not negatively affect fruit quality. In summary, the present results support the implementation of cold phytosanitary treatment at 1.7 °C for 15 days for the navel orange.

## 5. Conclusions

This study has documented the effect of cold phytosanitary treatment on *B. dorsalis* mortality and postharvest quality of navel orange at low temperatures. The results confirmed that 15 days at 1.7 °C could provide quarantine security for controlling *B. dorsalis* at an efficacy level of 99.9916%. Moreover, cold treatment did not negatively affect fruit quality. Therefore, this cold treatment showed potential as a commercial quarantine treatment for navel orange in international trade.

## Figures and Tables

**Table 1:** Mortality (mean ± SE) of *Bactrocera dorsalis* subjected to cold treatment at 1.7 °C in navel oranges.

Stage	No. Treated	Exposure (Days)						
		Control	1	3	5	7	9	11
**Eggs**	5600	22.5 ± 2.45	84.19 ± 1.76	99.19 ± 0.31	99.84 ± 0.16	100.0 ± 0.00	100.0 ± 0.0	100.0 ± 0.0
2 day-old larvae	3976	8.16 ± 3.09	22.98 ± 7.88	46.68 ± 4.50	90.73 ± 6.64	99.46 ± 0.29	100.0 ± 0.0	100.0 ± 0.0
4 day-old larvae	3991	7.87 ± 2.29	22.09 ± 5.32	46.34 ± 9.26	87.80 ± 5.60	97.59 ± 1.72	100.0 ± 0.0	100.0 ± 0.0
6 day-old larvae	3932	12.49 ± 11.75	48.55 ± 4.94	81.63 ± 6.50	94.77 ± 1.61	99.66 ± 0.34	100.0 ± 0.0	100.0 ± 0.0

**Table 2:** Probit and logit analysis of *Bactrocera dorsalis* mortality response to cold treatment at 1.7 °C.

Analyzing Model	Stage	Slope ± SE	LT_99_ (95% CL) (Days)	LT_99.9968_ (95% CL) (Days)
Probit	Eggs	0.62 ± 0.07	3.1 (2.6, 4.3)	5.8 (4.5, 8.6)
	2 day-old larvae	0.58 ± 0.03	7.1 (6.2, 8.8)	10.0 (8.4, 12.9)
	4 day-old larvae	0.47 ± 0.02	7.8 (6.7, 9.6)	11.3 (9.5, 14.6)
	6 day-old larvae	0.41 ± 0.02	6.4 (5.7, 7.3)	10.5 (9.2, 12.3)
Logit	Eggs	1.45 ± 0.19	3.0 (2.6, 3.6)	7.0 (5.8, 8.9)
	2 day-old larvae	1.07 ± 0.08	7.5 (6.3, 10.3)	12.9 (10.1, 19.6)
	4 day-old larvae	0.82 ± 0.04	8.4 (6.9, 11.7)	15.4 (12.0, 23.3)
	6 day-old larvae	0.78 ± 0.04	6.8 (5.9, 8.1)	14.2 (12.0, 17.7)

**Table 3:** Results of large-scale tests on second instar *Bactrocera dorsalis* larvae in navel oranges for 15 days at 1.7 °C.

Trial No.	Treated		
	No. of Fruit Infested	No. of Insects Treated	No. of Surviving Individuals
Control	18	4724	4445
1	48	13,136	0
2	48	8784	0
3	48	15,872	0
	Total	37,792	0
